# Effects of Different Drying Methods on the Structural Characteristics and Multiple Bioactivities of *Rosa roxburghii* Tratt Fruit Polysaccharides

**DOI:** 10.3390/foods13152417

**Published:** 2024-07-30

**Authors:** Qiuqiu Zhang, Sha Wu, Qinghua Dai, Peng Hu, Guangjing Chen

**Affiliations:** 1 College of Food Science and Engineering, Guiyang University, Guiyang 550005, China; zhangqq1248@163.com (Q.Z.); 15120336039@163.com (S.W.); 17785959192@163.com (Q.D.); 2 School of Pharmacy, Hunan Traditional Chinese Medical College, Zhuzhou 412012, China

**Keywords:** *Rosa roxburghii* Tratt, polysaccharides, drying method, physicochemical characteristics, antioxidant, protein glycation, α-glucosidase

## Abstract

Drying conditions significantly impact the compositions and microstructures of polysaccharides, leading to various effects on their chemical characteristics and bioactivities. The objective of this study was to investigate how different industrial drying techniques, i.e., hot air drying, infrared drying, microwave vacuum drying, and freeze drying, affect the structural properties and biological activities of polysaccharides extracted from *Rosa roxburghii* Tratt fruit (RRTP). Results revealed that these drying methods significantly altered the extraction yield, molecular weights, monosaccharide ratios, contents of uronic acid and total sugars, gelling properties, particle sizes, thermal stability, and microstructures of RRTPs. However, the monosaccharide composition and functional groups of polysaccharides remained consistent across the different drying techniques. Biological activity assays demonstrated that RRTPs, particularly those processed through microwave vacuum drying (MVD-RRTP), exhibited excellent anti-linoleic acid oxidation, robust anti-glycosylation effects, and significant α-glucosidase inhibition in vitro. The outcomes of this research demonstrate that microwave vacuum drying serves as an effective pre-extraction drying method for RRTPs, enhancing their biological activities. This technique is particularly advantageous for preparing RRTPs intended for use in functional foods and pharmaceuticals, optimizing their health-promoting properties for industrial applications.

## 1. Introduction

*Rosa roxburghii* Tratt (RRT), a member of the Rosaceae family native to southwest China, thrives on shrubs at altitudes ranging from 500 to 2500 m. Known locally in China as ‘Cili’, the robust, golden RRT fruit has long served as a dietary staple in southwest China, particularly in Guizhou Province [1]. Recent pharmacological research has revealed a spectrum of beneficial properties in RRT, including antioxidant, anti-tumor, anti-mutagenic, anti-diabetic, and anti-inflammatory effects [2,3,4]. These effects are attributed to its diverse natural active compounds. Several key active ingredients in RRT fruit have been identified, such as polysaccharides, organic acids, flavonoids, polyphenols, terpenoids, and essential oils, which endow it with significant potential for functional food development, dietary supplements, and pharmaceutical applications [5,6]. Polysaccharides, a pivotal natural component of RRT, have garnered considerable attention for their various biological activities. For instance, Chen et al. [3] highlighted the ability of RRT polysaccharides to inhibit metastasis and invasion of ovarian cancer cells, suggesting potential as a novel therapeutic approach for ovarian cancer. Additionally, Wu et al. [4] demonstrated that RRT polysaccharides exhibit notable inhibition of digestive enzyme activity and antioxidant capacity in vitro. Moreover, Wang et al. [7] found that the RRT polysaccharide (RTFP-3) simulates the digestion process in saliva and the gastrointestinal tract, with a significant portion reaching the large intestine where it enhances total short-chain fatty acid content, thereby modulating gut microbiota composition. Moreover, the efficacy of RRT polysaccharides can vary depending on the extraction method used. For example, ultrasound-assisted water extraction, as explored by Chen et al. [8], yielded polysaccharides with enhanced antioxidant capacity, leading to improved antioxidant enzyme activity (CAT, SOD, and GSH-Px) in serum and liver tissue observed in mouse experiments.

In Guizhou, RRT is economically cultivated across 1.76 million acres, yielding 66,000 tons of fresh fruit in 2019 [1]. Despite this substantial output, meeting rising demand presents challenges such as nutrient degradation and rapid decay due to the fruit’s high-water content and active respiration [9]. Effective drying technologies are essential for extending shelf life and facilitating further processing in RRT industries. Numerous drying methods are utilized in fruit processing and preservation, including hot air drying, infrared drying, freeze drying, vacuum drying, and microwave drying. These methods significantly influence the structure, physicochemical properties, and biological characteristics of polysaccharides, thereby impacting their antioxidant, hypoglycemic, and immune-regulatory properties [10]. For example, Ma et al. [11] observed that freeze-dried polysaccharides exhibited a rough, loose, and porous morphology, likely due to ice crystal formation and water sublimation disrupting the cellular structure. Microwave drying elevated the molecular weight of dandelion polysaccharides from 4.44 × 10^4^ Da (using freeze-drying) to 8.40 × 10^4^ Da. This increase is attributed to the higher temperatures involved in microwave drying, which disrupt the hydration layer of the polysaccharides, expose the hydroxyl groups on their chains, and lead to aggregation [12]. Similarly, studies on lotus leaf polysaccharides indicated that higher temperatures from hot air, microwave, and radio frequency drying methods reduced molecular weight compared to freeze and vacuum drying [13]. Notably, hot-air drying increased glucose content, reduced galactose content, and enhanced antioxidant capacity compared to freeze vacuum drying, altering the polysaccharide configuration [14]. Given these diverse effects, the selection of appropriate drying methods is critical for controlling the physicochemical and pharmacological quality of polysaccharides. Polysaccharides constitute significant chemical components of RRT and are inevitably affected during drying processes in food processing. However, the impact of drying technologies on the chemical composition, molecular weight, physicochemical structure, and biological activity of these active polysaccharides remains unclear.

Therefore, this study investigates four drying techniques applied to RRT, namely infrared radiation drying (IRD), hot-air drying (HD), freeze vacuum drying (FD), and microwave vacuum drying (MVD). It compares their effects on the physical and chemical attributes, such as the composition, molecular weight, rheological properties, and structural characteristics, of polysaccharides, as well as their biological properties, including resistance to linoleic acid peroxidation, anti-glycosylation activity, and in vitro inhibitory activity on α-glucosidase. The findings aim to enhance understanding of how different drying methods influence the structure and biological functions of RRT polysaccharides, thereby guiding the selection of optimal drying methods for producing hypoglycemic and anti-glycosylated agents in the functional food industry.

## 2. Materials and Methods

### 2.1. Materials and Chemicals

In September 2022, fresh RRT fruits were harvested from farmland in Longli, Guizhou Province, China (26°33′19.20″ N, 106°55′12.24″ E, 1380 m above sea level). These fruits were obtained from the same batch and initially stored at 4 °C before undergoing the drying process. Chemical standards, including standard monosaccharides, 3-phenylphenol, and trifluoroacetic acid (TFA), were sourced from TOKYO Chemical Industry Co., Ltd., Tokyo, Japan. Sodium hydroxide was obtained from Thermo Fisher Co., Waltham, MA, USA, while the pullulan polysaccharide calibration kit was procured from Agilent Co., Santa Clara, CA, USA. P-nitrobenzene α-D-pyran glucoside (PNPG) was acquired from Aladdin Biochemical Technology Co., Ltd. (Shanghai, China), and α-Glucosidase (S10050-100U) from Yeyuan Biotech Co., Ltd. (Shanghai, China). Aminoguanidine hydrochloride (AG) and acarbose water hydrate were purchased from Macklin Biochemical Co., Ltd. (Shanghai, China), and Bovine Serum Albumin V (BSA, A8020-100g) from Solarbio Technology Co., Ltd. (Beijing, China). All other chemical reagents and solvents used throughout the study were of analytical or chromatographic grade.

### 2.2. Drying Experiments

Fresh RRT fruits were prepared by washing and slicing them into 10–15 mm pieces. These slices were then evenly divided into four groups weighing 3.0 kg for different drying methods: IRD, HD, FD, and MVD. For the IRD group, RRT slices were placed in an infrared drying oven (DHG-9202-3, Changzhou Henglong Instrument Co., Ltd., Guangzhou, China) set to 1000 W. The slices were arranged in a single layer on drying plates and dried at 65 °C for 16 h. In the HD group, slices were evenly spread in an electric heating constant temperature drum wind drying oven (DHG-9240A, Shanghai Qixin Scientific Instrument Co., Ltd., Shanghai, China) and dried at 60 °C for 34 h. For the FD method, RRT slices were evenly distributed on a porous tray and dried in a BLK-FD-1 freeze dryer (Jiangsu BoLaiKe Instrument Co., Ltd., Changzhou, China) under a vacuum of 10 Pa. The drying process lasted 50 h and involved the following stages: pre-freezing at −50 °C for 2 h, followed by holding at 5 °C for 15 h, then adjusting to 10 °C for 10 h, increasing to 20 °C for another 10 h, reaching 30 °C for 5 h, and finally, maintaining at 45 °C for 8 h. In the MVD group, RRT slices were dried using an intelligent static vacuum microwave dryer (WBZ-10PLC, Guiyang Novel Microwave Industry Co., Ltd., Guiyang, China). The drying process occurred at 0.08 MPa over 75 min in an intermittent manner, with temperatures maintained between 40–50 °C. The drying procedure included stages of 1000 W for 20 min, 500 W for 35 min, 300 W for 15 min, and finally, 0 W for 5 min. Moisture content was monitored using a moisture meter, and all dried RRT fruit slices achieved approximately 6% moisture content on a wet basis after drying.

### 2.3. Preparation of RRT Polysaccharides

Four types of dried RRT fruit samples were ground and sieved through a 60-mesh sieve to obtain fruit powders. Each powder was soaked separately in petroleum ether, and 95% ethanol (*v*/*v*) for 24 h, and the resulting precipitate was collected and dried in a thermostatic blast drying incubator at 40 °C for 24 h. Subsequently, the powders from the IRD, HD, FD, and MVD samples were mixed with deionized water at a ratio of 1:20. The polysaccharides were then extracted using hot water extraction at 90 °C for 2 h. After extraction, the polysaccharide extract was centrifuged at 4000 rpm for 10 min to obtain the supernatant. The polysaccharide extract was concentrated to 1/4 of its volume using rotary evaporation, followed by deproteinization using Sevag reagent (chloroform to n-butanol ratio of 4:1, *v*/*v*) repeated five times. The polysaccharide precipitate was obtained by alcohol precipitation using four volumes of anhydrous ethanol and then lyophilized to obtain dried polysaccharides. The polysaccharide dry products were coded according to the corresponding drying technology: IRD-RRTP, HD-RRTP, FD-RRTP, and MVD-RRTP. The yield (%) of the RRTPs is calculated as follows:

(1) $$RRTPs\;yield\;(\%,\;w/w)=\frac{weight\;of\;dried\;RRTPs\;(g)}{weight\;of\;pretreated\;RRT\;fruit\;powder\;(g)}\times 100.$$

### 2.4. Structural Characterization of RRTPs

#### 2.4.1. Chemical Composition and Molecular Weight Determination

In the experimental method following Bradford, the protein content in the polysaccharide samples was quantified using bovine serum albumin as a standard reference [15]. The neutral polysaccharide content of RRTPs was determined using the phenol-sulfuric acid method [16]. To analyze the uronic acid content in RRTPs, the *m*-hydroxybiphenyl method was employed, with galacturonic acid serving as the standard reference [17].

The molecular weight distribution of RRTPs was assessed using high-performance gel permeation chromatography (HPGPC) on an Agilent 1260 chromatograph (Agilent Technologies, Santa Clara, CA, USA). The chromatograph was equipped with a TSKgel GMPWXL column (7.8 mm × 300 mm, Tosoh Biotech, Tokyo, Japan) maintained at 35 °C throughout the experiment. A mobile phase of 0.1 M NaNO_3_ was used, and the polysaccharide solution (5 mg/mL, 25 μL) was eluted at a flow rate of 0.6 mL/min over a period of 32 min. For molecular weight calibration, a series of pullulan standards with known molecular weights (Mw), including 6.3, 9.8, 22.0, 49.7, 216, and 334 kDa, were employed to generate a standard calibration curve.

#### 2.4.2. Monosaccharide Composition

High-performance anion-exchange chromatography (HPAEC) with a pulsed amperometric detector and a Dionex Carbopac PA20 column (3 mm × 150 mm, 5 μm) was used to analyze the monosaccharide composition of the polysaccharides. Initially, polysaccharides (6 mg) were hydrolyzed using 3 M trifluoroacetic acid (3 mL) at 121 °C for 3 h. Trifluoroacetic acid was subsequently removed by rotary evaporation, and any residual trifluoroacetic acid was washed away with methanol [18]. The resulting residues were dissolved in deionized water and filtered through a 0.22 μm membrane filter. During analysis, an automatic injection of 20 μL was made into the HPAEC system (ICS 6000, Thermo Fisher, Waltham, MA, USA), which employed a gradient elution with a flow rate of 0.6 mL/min using four liquid phases: A (ultrapure water), B (0.02 M NaOH), C (0.5 M NaOAc), and D (0.1 M NaOH). The gradient elution profile was as follows: starting with 95% A and 5% B, transitioning to 55% A, 20% B, and 25% C from 16.01 to 38 min, and finally, adjusting to 20% A and 80% B from 38.01 to 55 min. Monosaccharide standards used for calibration included fucose (Fuc), rhamnose (Rha), arabinose (Ara), glucose (Glc), galactose (Gal), mannose (Man), xylose (Xyl), galacturonic acid (GalA), and glucuronic acid (GlcA).

#### 2.4.3. Triple-Helical Structure and Fourier Transform Infrared (FTIR) Spectroscopy

To explore the triple-helical structure of RRTPs, Congo red was employed following a previously described method [19]. A solution of RRTPs (2 mL, 2.0 mg/mL) was mixed with Congo red solution (2 mL, 80 μM). Subsequently, 1 M NaOH solution was added incrementally to the mixture to achieve final NaOH concentrations ranging from 0.05 to 0.50 mol/L. After each addition of NaOH, the mixture was equilibrated for 10 min at room temperature (25 °C), and then the maximum absorption wavelength (λmax) was measured using a multifunctional microplate reader (Multiskan SkyHigh, Thermo Scientific, Waltham, MA, USA) within the wavelength range of 400–600 nm.

The infrared spectra of RRTPs were obtained using an FT-IR spectrophotometer (Spectrum Two, PerkinElmer Co., Waltham, MA, USA), following established protocols from the literature [20]. Polysaccharide samples were mixed with potassium bromide at a ratio of 1:100 mg. Spectra of RRTPs were recorded at a resolution of 4 cm^−1^ over a spectral range from 400 to 4000 cm^−1^. To assess the degree of esterification (DE) of each RRTP fraction, the band areas at 1700–1750 cm^−1^ (*A*_1740_, representing esterified uronic acids) and 1600–1630 cm^−1^ (*A*_1620_, indicative of free uronic acids) were analyzed. The DE of RRTPs was calculated using the following equation [21]:

(2) $$\mathrm{DM}\;=\frac{A_{1740}}{A_{1740}+A_{1620}}\;.$$

#### 2.4.4. Scanning Electron Microscopy (SEM) and X-ray Diffraction (XRD)

The dried polysaccharides were mounted on an aluminum holder and coated with a thin layer of gold using a sputter coater under reduced pressure. SEM images were then captured using an SEM instrument (EVO-18, ZEISS, Oberkochen, Germany) at magnifications of 200×, 2000×, and 20,000×, respectively. To determine the crystal structure of the RRTPs, XRD analysis was performed using a Bruker instrument (D8 Advance, Bruker, Bremen, Germany). The diffraction angular range was set from 10° to 80° (2θ angle range), with a scan speed of 5°/min.

#### 2.4.5. Particle Size and Potential

The particle size and zeta potential of RRTP solutions were determined using a nanosize and potential analyzer (NS-90Z, Malvern Zetasizer Co., Ltd., Worcestershire, UK). Each sample was prepared at a concentration of 2 mg/mL, and measurements were conducted in triplicate at a temperature of 25 °C.

#### 2.4.6. Differential Scanning Calorimetry (DSC)

Differential scanning calorimetry (DSC-4000, PerkinElmer Co., USA) was employed to investigate the thermal behavior and analyze the thermal stability of RRTPs. Each analysis involved placing two crucibles on the sample plate simultaneously: one containing 5 mg of the polysaccharide sample and the other serving as an empty reference crucible. The temperature range for analysis was set from 45 °C to 445 °C. The heating rate during the experiment was 10 °C/min, and nitrogen gas flowed at a rate of 50 mL/min throughout the heating process.

### 2.5. Rheological Measurement

#### 2.5.1. Steady Flow Behavior

The viscosity of RRTPs at different concentrations (0.5, 1.0, 1.5, 2.0, and 2.5 wt%) was analyzed using an MCR 302e hybrid rheometer (Anton Paar, Graz, Austria) equipped with parallel plate geometry (50 mm diameter, 1.0 mm gap) [22]. Steady shear measurements were conducted over a shear rate range from 0.1 to 1000 s^−1^ at 25 °C. The relationship between apparent viscosity and shear rate was determined through continuous shear testing. Prior to each test, samples were equilibrated at 25 °C for 1 min. All measurements were performed in triplicate.

#### 2.5.2. Frequency Sweep

Before conducting dynamic oscillation frequency measurements, the linear viscoelastic region (LVR) was determined using a 50 mm parallel plate geometry with a 1 mm gap. The LVR was systematically identified based on the storage modulus (G′) and loss modulus (G″), with the strain amplitude varied from 0.1% to 120%. For dynamic oscillatory tests, a solution of RRTPs at a concentration of 25 mg/mL was used. The G′ and G″ were measured under experimental conditions involving 1% strain amplitude and frequencies ranging from 1 to 100 rad/s (ω).

### 2.6. Antioxidant Activity in a Linoleic Acid System

The anti-oxidative effect of RRTPs was evaluated using the thiobarbituric acid (TBA) method [23]. The linoleic acid system was prepared by mixing 4.1 mL of linoleic acid-absolute ethanol solution (5%, *v*/*v*), 10 mL of phosphate buffer (0.2 M, pH 7.4), 1 mL of FeSO_4_·7H_2_O (4 mM), and distilled water. Various concentrations of RRTPs solution (0.125, 0.25, 0.5, 1.0, 2.5, and 5 mg/mL) were added to the system and allowed to react at 40 °C for 24 h. The reaction was stopped with 1 mL of 25% (*v*/*v*) trichloroacetic acid (TCA) solution, followed by the addition of 2 mL of 0.67% (*w*/*v*) TBA solution. After heating in boiling water for 15 min and cooling with 4 mL of *n*-butanol, absorbance readings were taken at 532 nm. The inhibition of linoleate peroxidation was calculated using the following formula:

(3) $$\mathrm{Inhibition}\;\mathrm{rate}\;(\%)=(1-\frac{A_{S}}{A_{B}})\;\times 100,$$

where *A_S_* represents the absorbance of the sample reaction solution, and *A_B_* represents the absorbance of the control solution without RRTPs.

### 2.7. In Vitro α-Glucosidase Activity Assay

#### 2.7.1. Inhibitory Effect on α-Glucosidase

The α-glucosidase inhibitory activity of RRTPs was evaluated with slight modifications to a standard method [24]. RRTPs were prepared at concentrations ranging from 0.5 to 8.0 mg/mL, along with α-glucosidase (0.5 U/mL) and p-nitrophenyl-α-D-glucopyranoside (pNPG, 5 mM) in phosphate-buffered saline (100 mM, pH 6.9). Each reaction vial contained 100 μL of RRTPs and 100 μL of α-glucosidase, which were incubated at 37 °C for 10 min. Subsequently, 100 μL of pNPG were added and further incubated at 37 °C for 20 min. The reaction was halted with 1 mL of sodium carbonate (1 M). Acarbose was used as the positive control. Absorbance at 405 nm was measured using a multifunctional microplate reader. Finally, a linear regression analysis determined the concentration-dependent inhibition rate and the semi-inhibitory concentration values of acarbose and RRTPs were calculated from this regression equation. The inhibition rate of α-glucosidase was calculated as follows:

(4) $$\mathrm{Inhibition}\;\mathrm{rate}\;(\%)=\left(1-\;(\frac{A_{S}-A_{B1}\;}{A_{B2}})\;\right)\times 100,$$

where *A_S_* is the absorbance of the sample reaction solution, *A_B_*_1_ is the absorbance of the mixture of pNPG solution and RRTPs without enzyme addition, and *A_B_*_2_ is the absorbance of the mixture of pNPG and enzyme without RRTPs.

#### 2.7.2. Inhibitory Kinetics of α-Glucosidase

To determine the inhibition type of α-glucosidase by RRTPs, kinetic properties were analyzed using Lineweaver–Burk double-reciprocal plots based on Michaelis–Menten kinetics (Equation (5)) [25]. From these plots, the Michaelis–Menten constant (*K_m_*) and the maximum velocity (*V_max_*) of the enzymatic reaction were determined. Various concentrations of RRTPs (0, 1.0, 4.0, and 8.0 mg/mL) and pNPG substrates (1.0, 2.0, 3.0, and 4.0 mM) were used. The enzyme–inhibitor dissociation constant (*K_i_*) was determined from the *x*-axis intercept of the Lineweaver–Burk plot and the inhibitor–enzyme–substrate dissociation constant (*K_is_*) was determined from the *y*-axis intercept [26].

(5) $$\frac{1}{v}=\frac{1}{V_{max}}(1+\frac{\left[S\right]}{K_{is}})+\frac{K_{m}}{V_{max}}\frac{1}{S}(1+\frac{\left[S\right]}{K_{i}})\;.$$

#### 2.7.3. Fluorescence Quenching

Specific quantities of α-glucosidase (1.5 mL at a concentration of 0.75 U/mL) were incubated with varying concentrations of RRTPs (1.0, 2.0, 3.0, 4.0, 5.0, 6.0, and 7.0 mg/mL) for 10 min at 310.15 K. The fluorescence of α-glucosidase was measured using a fluorescence spectrophotometer (F-320, Gangdong Technology Co., Ltd., Tianjin, China). The spectrophotometer settings were as follows: excitation and emission grating slits were set at 5 nm, the excitation wavelength was 280 nm, and the emission wavelength was between 300–400 nm [27]. To analyze the fluorescence quenching mechanism of α-glucosidase by RRTPs, the bimolecular quenching constant (*K_q_*) and the Stern–Volmer quenching constant (*K_sv_*) were determined using the Stern–Volmer equation (Equation (6)). Subsequently, the fluorescence quenching data were further assessed using the double logarithmic plot equation (Equation (7)) to calculate the binding constant (*K_a_*) and the number of binding sites (*n*).

(6) $$\frac{F_{0}}{F}=1+K_{q}\tau_{0}\left[Q\right]=1+K_{sv}\left[Q\right],$$

(7) $$\log\frac{F_{0}-F}{F}=n\log\left[Q\right]+\log K_{a}\;,$$

where *F* and *F*_0_ denote the fluorescence intensity with and without RRTPs, respectively; *Kq* denotes the quenching constant of α-glucosidase; τ_0_ signifies the lifetime of the fluorophore for α-glucosidase, set at 10^−8^ s; [Q] represents the concentrations of RRTPs; *Ka* denotes the binding constant between RRTPs and α-glucosidase; *n* indicates the number of binding sites [28].

### 2.8. In Vitro Anti-Glycation Assay

The non-enzymatic glycosylation system involving bovine serum albumin (BSA) and fructose was established following the methods described by Zhang et al. [29], with some adaptations. This model was used to evaluate the inhibitory effects of RRTPs on non-enzymatic protein glycosylation in vitro. Specifically, 5 mL BSA (20 mg/mL), 5 mL glucose solution (500 mmol/L), and 5 mL RRTPs solution (0.2 mg/mL) were combined to form the sample group. Two control sets were prepared: a blank control where the sample solution was replaced with 0.2 M pH 7.4 phosphate buffer and a positive control where the sample solution was substituted with aminoguanidine (AG) at an equivalent concentration to RRTPs. The mixtures were then incubated for 24 h at 50 °C to facilitate the three stages of glycation, as outlined in the experimental protocols detailed by Dou et al. [30].

#### 2.8.1. Determination of Fructosamine Concentration

To determine the fructosamine concentration, 40 μL of the reaction solution were mixed with 320 μL of ultrapure water and added to 1.6 mL of NBT solution (0.3 mM in sodium carbonate buffer, pH 10.35). The mixture was incubated at 25 °C for 15 min, and the absorbance was measured at 530 nm. The same procedures were applied to both blank and positive control samples. The inhibition rate of fructosamine (%) was calculated using the following formula:

(8) $$Inhibition\;rate\;(\%)=(\frac{A_{C}-A_{S}}{A_{C}})\times 100,$$

where *A_S_* corresponds to the absorbance of the glycosylation system post-sample addition, while *A_C_* signifies the absorbance of the glycosylation system in the blank control group.

#### 2.8.2. Determination of α-Dicarbonyl Compounds

For α-dicarbonyl compounds, 0.4 mL of the reaction solution were mixed with 0.2 mL of Girard-T solution (500 mM) and 3.4 mL of sodium formate solution (500 mM, pH 2.9). The mixture was incubated for 1 h at 37 °C, and the absorbance was read at 290 nm. The inhibition rate of α-dicarbonyl compounds was calculated using Equation (8).

#### 2.8.3. Determination of Fluorescent AGEs

For fluorescent AGEs determination, fluorescence intensity was measured using a spectrophotometer (F-320, Gangdong Technology Co., Ltd., Dongguan, China). A mixture of 2 mL of PBS solution (0.2 M, pH 7.4) and 60 μL of glycosylation solution was used. The instrument settings were an excitation wavelength of 370 nm and an emission wavelength of 450 nm. The inhibition rate of fluorescent AGEs was determined using the following formula:

(9) $$Inhibition\;rate\;(\%)=(\frac{F_{C}-F_{S}}{F_{C}})\times 100$$

The fluorescence intensity *F_S_* indicates the level after sample addition, while *F_C_* refers to the intensity in the blank control group for the glycosylation system.

### 2.9. Statistical Analysis

The data were expressed as mean ± standard deviation (SD) from three replicates per assay. Statistical significance was assessed using one-way ANOVA with SPSS 16.0 software. Differences were considered statistically significant at *p* < 0.05. The data were graphically depicted using OriginPro 2021 Learning Edition, developed by OriginLab Corp., based in Northampton, MA, USA.

## 3. Results and Discussion

### 3.1. Effects of Different Drying Methods on the Physicochemical Characteristics of RRTPs

#### 3.1.1. The Yield and Chemical Compositions of RRTPs

The yields of RRTPs from IRD, HD, FD, and MVD were 2.96%, 3.23%, 3.75%, and 4.38%, respectively (Table 1). This indicates that MVD and FD resulted in higher RRTP yields, consistent with previous findings (3.57%) [31]. These results are in agreement with Fu et al.’s observation that microwave-dried loquat leaves demonstrate superior polysaccharide extraction efficiency [32]. The higher yield from MVD is likely due to the disruptive effects of microwave-generated high vapor pressure and temperature, effectively breaking down cell wall polymers to enhance polysaccharide extraction. Moreover, under vacuum conditions, the expansion of gaseous substances forms a porous structure that improves permeability, allowing extracting agents to penetrate tissues or cells more efficiently during extraction [33]. In contrast, lower yields observed with HD and IRD may be attributed to severe fruit shrinkage and the formation of a hardened surface (“case hardening”) during hot air and infrared drying processes [34].

Table 1 illustrates that the total sugar content in RRTP fruit extracts ranged from 57.47% to 68.56%, with polysaccharides as the predominant component. Among the four drying methods, MVD-RRTP exhibited the highest total neutral sugar content, followed by FD-RRTP, IRD-RRTP, and HD-RRTP. Similarly, the uronic acid content in RRTPs followed a corresponding trend, with percentages of 22.16%, 18.09%, 15.78%, and 15.46% for MVD, FD, IRD, and HD drying methods, respectively. Despite thorough deproteinization and dialysis, residual protein levels ranged from 1.57% to 2.82% in the samples, likely stemming from protein–polysaccharide complexes present in the extracted polysaccharides. The higher concentrations of neutral sugar and uronic acid in MVD-RRTP are attributed to reduced enzymatic activity during microwave vacuum drying, particularly enzymes like polysaccharide hydrolase and glucuronidase. Previous research has linked enzyme activity to neutral sugar and uronic acid contents, with optimal enzyme activity occurring between 50 °C and 80 °C [35]. The lower temperatures and reduced oxygen levels during microwave vacuum drying likely suppressed enzyme activity, leading to higher retention of neutral sugar and uronic acid. In addition to enzymatic effects, thermal and oxidative processes also played pivotal roles [36]. This could explain why IRD-RRTP and HD-RRTP exhibited lower retention of neutral sugar and uronic acid, as both methods involve higher temperatures and increased oxygen exposure.

#### 3.1.2. Molecular Weights and Constituent Monosaccharides of RRTPs

**Figure 1:**
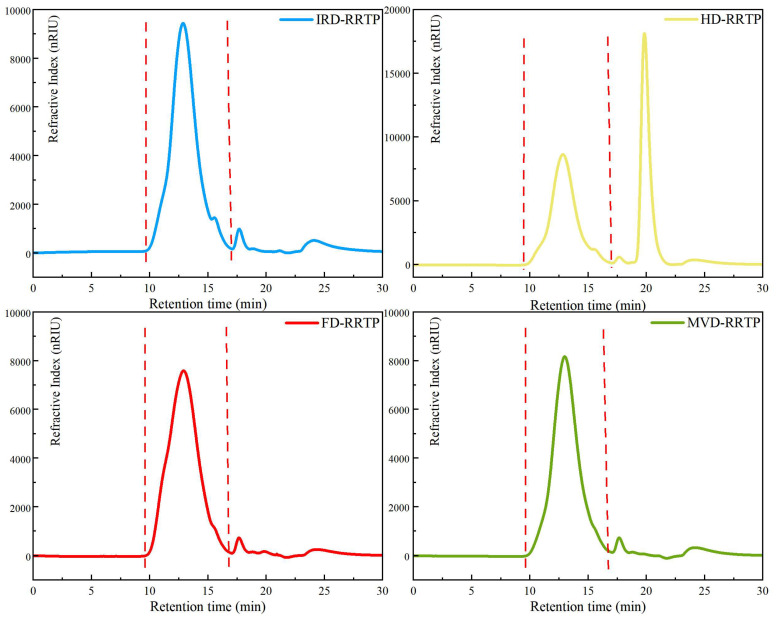
HPGPC chromatograms of RRTPs dried by different methods.

The HPGPC chromatograms and molecular weight parameters of RRTPs are presented in Figure 1 and Table 1, respectively. To quantitatively compare the polysaccharides, the weight-average (Mw), number-average (Mn), and polydispersity (Mw/Mn) of the four RRTPs were analyzed. It is evident that different drying methods yielded polysaccharides with varying molecular weights. MVD-RRTP exhibited the smallest Mw at 145.68 kDa, whereas FD-RRTP showed the largest Mw at 196.72 kDa. The impact of drying processes on the molecular weight distribution of polysaccharides from different sources has been extensively documented. Microwave energy, for example, has been reported to induce polysaccharide degradation due to shear forces, leading to main chain breakage and thermal degradation–hydrolysis [37,38]. This phenomenon likely explains the lower molecular weight observed in MVD-RRTP. Similar reductions in molecular weight following MVD treatment have been observed in studies on hawthorn and orange peel polysaccharides [38,39]. In contrast, FD typically preserves polysaccharide molecular chains without generating high temperatures, thereby avoiding thermal degradation and maintaining molecular weight [40]. This is consistent with the higher molecular weight observed in FD, as reported in studies on mulberry leaves [11]. The molecular size distribution of RRTPs was broad across all drying methods, indicating high dispersion regardless of the method employed (Table 1). The polydispersity indices of the four RRTPs were 9.15 (IRD), 9.73 (FD), 9.38 (HD), and 7.95 (MVD), with FD-RRTP exhibiting the widest distribution. This reflects FD-RRTP’s uniform distribution without thermal aggregation, resulting in broad molecular weight distributions.

**Figure 2:**
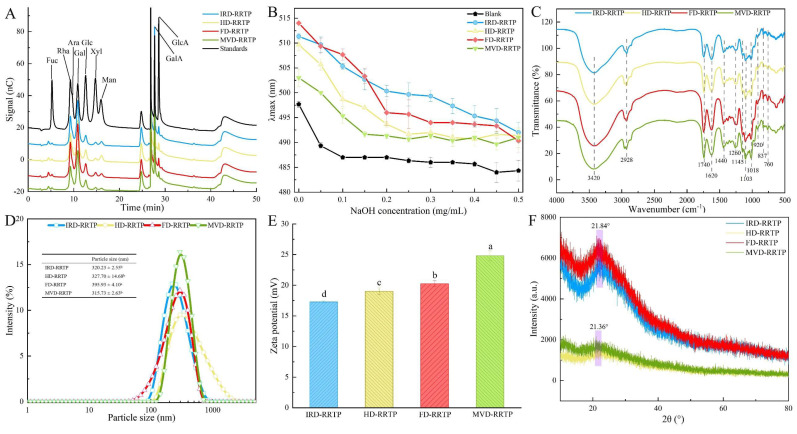
(**A**) HPAEC chromatograms of RRTPs; (**B**) effect of RRTPs on the absorbance of Congo red at different NaOH concentrations; (**C**) FT-IR spectra of RRTPs; (**D**) particle size distribution curves of RRTPs; (**E**) ζ-potential of RRTPs; (**F**) XRD spectra of RRTPs. Values for particle size and ζ-potential with no letters in common are significantly different (*p* < 0.05).

Figure 2A illustrates the monosaccharide composition and content plots of the four RRTP samples. Table 1 indicates that the four RRTPs primarily contained Rha, Ara, Gal, and GalA in varying molar ratios. MVD-RRTP exhibited the highest GalA content (33.79%), while FD-RRTP had the highest Gla content (39.24%), with the other three RRTPs ranging from 34.58% to 35.69%. The prevalence of galacturonic acid suggests that the RRTP samples are acidic polysaccharides, likely classified as pectin polysaccharides [41]. Comparatively, previous studies on RRT polysaccharides (PR-1) have emphasized galacturonic acid, glucose, galactose, and arabinose, differing from our findings [42]. These discrepancies in monosaccharide composition among RRTPs may arise from variations in raw material sources, extraction methods, and isolation procedures. The diverse molar ratios of monosaccharides among the polysaccharides likely stem from different drying techniques that can alter monosaccharide conformations. Pectic polysaccharides are classified into “smooth” and “hairy” regions. The former refers to the HG domain, comprising partially methylated and acetylated galacturonic acid, while the latter includes RG-I, rhamnogalacturonan-II (RG-II), and xylogalacturonan (XGA) domains with branches [43]. All four RRTPs had RG-I % values above 50%, indicating richness in Ara or Gal side chains [44], with HD-RRTP (79.53%) exhibiting the highest RG-I%. This prevalence suggests a multibranched structure in all RRTPs [45]. The Rha/GalA ratio reflects RG-I’s contribution to the pectin structure, while the (Ara + Gal)/Rha ratio indicates the average length of RG-I side chains. Rha/GalA ratios for the four RRTPs ranged from 0.48 to 0.62, with RG-I regions exceeding 50%, indicating a rich presence of galactose and arabinogalactose side chains attached to a short RG-I backbone [46]. MVD-RRTP exhibited the longest average RG-I side chain length (2.75). Additionally, the GalA/(Fuc + Rha + GlcA + Ara + Gla + Xyl) molar ratio, reflecting pectin’s linearity [47], showed that MVD-RRTP had a higher proportion of side chains (0.56) compared to IRD-RRTP, HD-RRTP, and FD-RRTP. These findings suggest that while polysaccharides from the four drying methods have similar monosaccharide compositions, drying processes can influence the proportions of the pectin region structure, RG-I side chain structures, and the linear nature of RRTPs.

#### 3.1.3. Triple-Helical Structure and FT-IR Analysis of RRTPs

In mildly alkaline conditions, polysaccharides with triple helical structures can bind with Congo red, forming complexes that show a redshift in the maximum absorption wavelength compared to a solo Congo red solution. However, under strong alkaline conditions, this triple helical structure is disrupted, leading to a reduced redshift in the Congo red–polysaccharide complex [48]. To examine the triple-helix configuration of RRTPs, Congo red assays were conducted. Figure 2B illustrates the maximum absorption wavelength (λmax) and the difference between λmax and the blank control (λmax–λBlank) for the Congo red RRTPs complex across various NaOH concentrations. The absence of a redshift in λmax with increasing sodium hydroxide concentration suggested that the complexes did not undergo any significant shift in maximum absorption wavelength. This lack of a redshift indicated that RRTPs do not possess a triple-stranded helical structure, supporting findings from similar studies on RRTPs [49].

Figure 2C displays the FT-IR spectra of the four RRTPs across the wavelength range of 4000–500 cm^−1^, revealing uniformity among the characteristic functional groups despite variations in drying methods. A pronounced, broad absorbance band at 3420–3431 cm^−1^ identifies the characteristic peak of the -OH group. The absorption at 2925–2930 cm^−1^ corresponds to the C–H stretching vibrations of the methyl group [50]. Notably, absorption peaks at 1740 cm^−1^ and 1620 cm^−1^ in all samples indicate the presence of esterified and free carboxyl groups (-COOR), respectively, suggestive of uronic acid content [12]. Additionally, bands at 920 cm^−1^ and 760 cm^−1^ are characteristic of D-glucose in its pyranose form, and peaks at 830 cm^−1^ and 837 cm^−1^ suggest α-type glycosidic linkages. These findings align with previous research on RRTPs [49] and confirm that sugar ring structures and glycosidic bonds in RRTPs are consistent across different drying technologies. The FT-IR method also enables the determination of the degree of esterification (DE) by measuring the peak areas corresponding to free and esterified carboxyl groups [50]. DE values detailed in Table 1 indicate that FD-RRTP and MVD-RRTP exhibited higher DE compared to IRD-RRTP and HD-RRTP. The lower DE values in IRD-RRTP and HD-RRTP can be attributed to the gradual temperature rise during processing, which likely activates native pectic esterases, leading to pectin demethylation [38]. With DE values exceeding 50%, the extracted pectin from all samples qualifies as high methoxylated pectin, which is more resistant to degradation by endo-α-1,4-polygalacturonase and remains stable under high temperatures [46], enhancing its utility in various food processing applications.

#### 3.1.4. Particle Size and Zeta-Potential Analysis of RRTPs

Particle diameter indicates the aggregation degree of polysaccharide molecules in solution, serving as an indirect reflection of their molecular weight [51]. As demonstrated in Figure 2D, all four RRTPs showed a single peak with relatively uniform particle size distributions. Specifically, the particle sizes for IRD-RRTP, HD-RRTP, FD-RRTP, and MVD-RRTP were 320.23, 327.70, 395.93, and 315.73 nm, respectively. FD-RRTP exhibited the largest particle size, while MVD-RRTP had the smallest, aligning with the results from molecular weight determinations. Figure 2E presents the zeta-potential of RRTPs. The zeta potentials for IRD-RRTP, HD-RRTP, FD-RRTP, and MVD-RRTP were −17.27, −20.23, −19.00, and −24.80 mV, respectively. These values indicate that the RRTP particles carry negative charges, primarily due to the presence of uronic acids. This observation aligns with the physicochemical property results of RRTPs. A higher absolute value of zeta potential indicates greater system stability, whereas a lower value suggests reduced stability.

#### 3.1.5. XRD and SEM Examination of RRTPs

XRD analysis is employed to determine the crystalline or amorphous characteristics of polysaccharides. Figure 2F shows that no sharp or strong absorption peaks were observed between 10° and 80° in any RRTP samples, indicating their amorphous nature. Instead, a subdued, broad peak around 20° was evident across all samples. Notably, the XRD patterns of HD-RRTP and MVD-RRTP showed significantly lower intensity compared to other samples, suggesting a reduced level of crystallinity. This indicates that hot drying and microwave vacuum drying may particularly influence the structural properties of polysaccharides, rendering them more amorphous.

**Figure 3:**
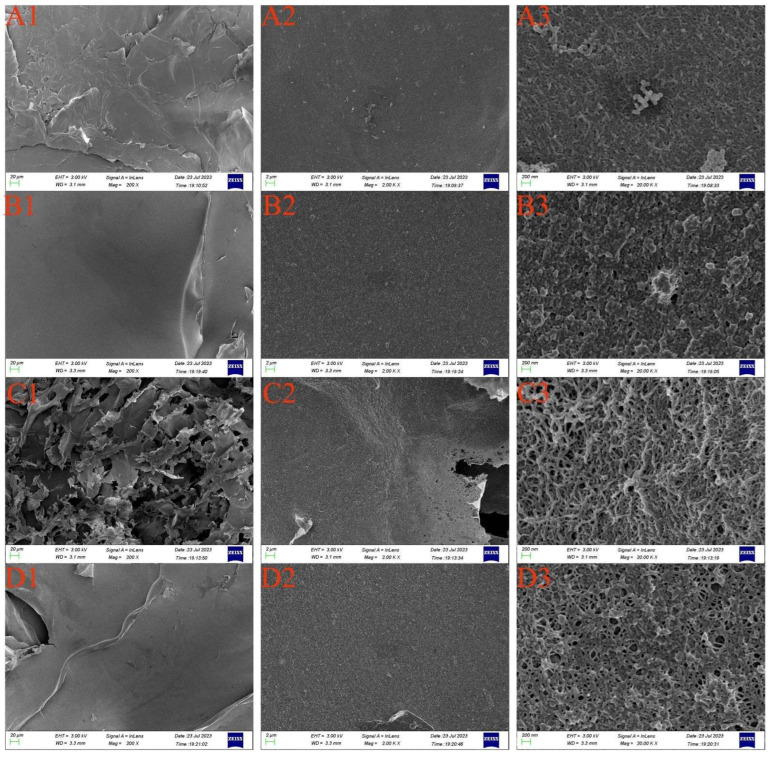
Scanning electron micrographs of RRTPs: IRD-RRTP (**A**), HD-RRTP (**B**), FD-RRTP (**C**), and MVD-RRTP (**D**). Each sample is shown at three magnifications: 200× (**1**), 2.0 k× (**2**), and 20.0 k× (**3**).

Figure 3 presents the surface morphology of RRTPs as observed through SEM at magnifications of 200×, 2.00 k×, and 20.00 k×. Each RRTP sample exhibits distinct size and shape characteristics. Under 200× magnification, FD-RRTP uniquely displays numerous irregular and fragmented structures, unlike the lamellar structures observed in the other three samples. This variation is attributed to the freeze-drying process, where pre-freezing leads to ice crystal formation that disrupts cell structures more severely compared to other drying methods [52]. At 2.00 k× magnification, IRD-RRTP and HD-RRTP present more compact structures in contrast to the porous structures of FD-RRTP and MVD-RRTP. The microstructure in HD-RRTP appears shrunken and tightened as a result of the temperature gradient and water movement during hot air drying. Infrared drying involves rapid penetration of infrared energy, causing internal moisture to accumulate rather than evaporate promptly, leading to the cross-linking of polysaccharides into dense structures with irregular protrusions [34]. At the higher 20.00 k× magnification, all RRTPs display a tight and regular banded network. However, HD-RRTP is characterized by a smooth, tightly packed surface with irregularly distributed beads. In contrast, FD-RRTP and MVD-RRTP show a more open and net-like surface composed of coralline and rod-like structures. These observations confirm that different drying methods result in distinct surface topographies and appearances in RRTPs, which are likely linked to variations in their physicochemical properties and antioxidant activities [53].

#### 3.1.6. DSC Analysis of RRTPs

DSC was employed to assess the endothermic and exothermic transformations occurring as temperature increased in RRTPs. The results, depicted in Figure S1 and summarized in Table 2, showed that all RRTP samples exhibited a pronounced endothermic peak between 121.50–148.72 °C, attributed to dehydration, loss of peripheral polysaccharide chains, or dehydroxylation reactions [54]. Additionally, an exothermic peak was observed across samples, indicating heat release associated with the decomposition or oxidative degradation of the polysaccharides [55]. This analysis also provided insights into the glass transition temperature (*Tg*) and degradation enthalpy (Δ*Hg*) of the samples, with *Tg* reflecting their thermal stability. Consistent *Tg* values suggest similar thermal stability among the RRTPs. The Δ*Hg* values recorded were 30.18 J/g for IRD-RRTP, 5.03 J/g for HD-RRTP, 21.67 J/g for FF-RRTP, and 3.21 J/g for WD-RRTP, with notably low Δ*Hg* for MVD-RRTP indicating a highly ordered molecular arrangement [46]. Furthermore, the broad exothermic peak for FD-RRTP suggests a wide molecular weight distribution [56], consistent with its molecular weight characteristics. All samples exhibited an exothermic peak of around 258 °C, highlighting their robust thermal stability. This characteristic is crucial for food industry applications, where materials with superior thermal stability are essential to preserve the nutritional quality of food during thermal processing.

### 3.2. Rheological Properties of RRTPs

#### 3.2.1. Apparent Viscosity

**Figure 4:**
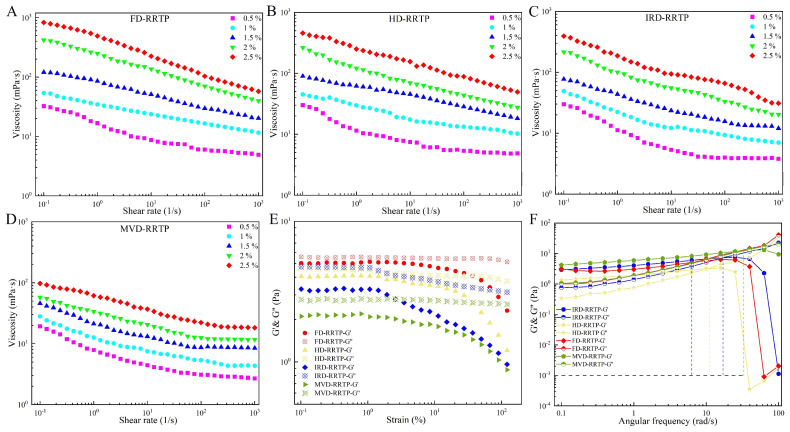
Rheological properties of RRTPs. (**A**–**D**) The apparent viscosities of RRTPs as a function of shear rate; (**E**) strain sweeps of RRTPs; (**F**) storage modulus (G′, solid symbols) and loss modulus (G″, open symbols) as a function of angular frequency ω for RRTPs.

The flow behavior of diluted RRTP solutions was studied at 25 °C. Figure 4A–D displays the steady shear flow curves for RRTP solutions across concentrations from 0.5% to 2.5% (*w*/*v*). Across a shear rate range of 0.01–1000 s^−1^, the viscosity of all four samples decreased with increasing shear rate, though the rate and extent of this decrease varied among the samples. Notably, FD-RRTP exhibited the highest viscosity, likely due to its higher molecular weight. In contrast, MVD-RRTP had a lower apparent viscosity compared to other RRTPs, potentially resulting from microwave processing, which may break the pectin chains, reducing molecular mass and decreasing the entanglement and interaction between pectin molecules [38]. The observed reduction in viscosity with increasing shear rate demonstrates the shear-thinning behavior of RRTP aqueous solutions. This behavior stems from the alignment of polymer molecular chains along the flow direction. As the shear rate increases, the degree of polymer chain orientation rises, reducing viscosity.

#### 3.2.2. Oscillatory Measurements

Polysaccharides exhibit viscoelastic properties, displaying both solid and fluid characteristics. The storage modulus (G′) indicates elasticity, while the loss modulus (G″) represents viscosity. Evaluating these moduli within the linear viscoelastic region (LVR) is crucial to determine the predominant behavior of a material. Figure 4E exhibits the LVR of the four RRTPs. A constant strain of 1% was chosen for G′ and G″. As shown in Figure 4F, at lower frequencies, where G′ exceeded the loss modulus G″, elastic properties predominate, suggesting the formation of a gel-like structure within the solution. As the frequency increases, G′ and G″ intersect at a crossover point, marking a transition in viscoelastic behavior and signaling the onset of elastic dominance or the approach to a gel state. Beyond this point, as the frequency continues to rise and G′ falls below G″, elasticity diminishes, indicating the breakdown of the gel-like structure. For the RRTPs derived from various drying methods, FD-RRTP, HD-RRTP, IRD-RRTP, and MVD-RRTP, the crossover points occurred at angular frequencies ranging from 6.31 to 23.16 rad/s. This variation implies that the different drying methods significantly affect the viscoelastic properties of RRTPs. Notably, the crossover point for MVD-RRTP occurred at a higher frequency than the other RRTPs, indicating superior gelling properties and enhanced system stability. This characteristic potentially makes MVD-RRTP especially suitable for food processing applications where stability is crucial [57].

### 3.3. Antioxidant Activity in a Linoleic Acid System

**Figure 5:**
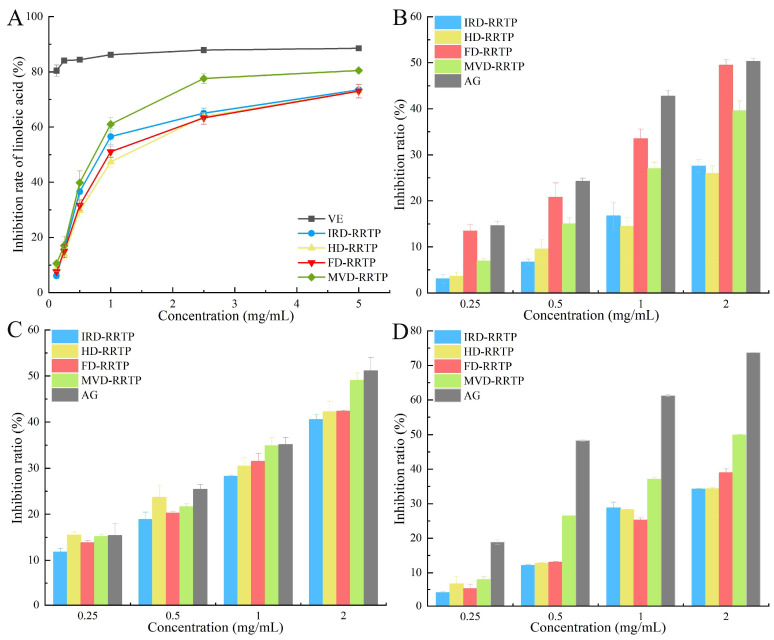
(**A**) Anti-linoleic acid oxidation inhibitory activity of RRTPs; (**B**) fructosamine inhibition of RRTPs; (**C**) α-dicarbonyl compounds inhibition of RRTPs; (**D**) AGEs inhibition of RRTPs.

In the presence of light, heat, and oxygen, oils can undergo rancidity, decomposing into aldehydes, acids, and other compounds. Monitoring oxides that form during oxidation is a standard method for assessing oil oxidation [58]. Malondialdehyde (MDA), a three-carbon dialdehyde produced by oil oxidation, is widely used as a marker for lipid oxidation [59]. The MDA content is quantified using the TBA method, where MDA reacts with 2-thiobarbituric acid (TBA) to form a red chromogen MDA-TBA, detectable at 532 nm with a spectrophotometer [60]. Figure 5A illustrates the inhibitory effects of different RRTPs on linoleic acid oxidation activity. The results showed that all four RRTPs displayed inhibitory effects on linoleic acid oxidation in a concentration-dependent manner. MVD-RRTP demonstrated the most potent inhibition (IC_50_ of 0.86 ± 0.04 mg/mL), followed by IRD-RRTP (IC_50_ of 1.20 ± 0.10 mg/mL), FD-RRTP (IC_50_ of 1.33 ± 0.08 mg/mL), and HD-RRTP (IC_50_ of 1.40 ± 0.08 mg/mL). These results underscored MVD-RRTP’s superior resistance to linoleic acid oxidation among the drying methods tested, possibly due to its lower molecular weight and higher uronic acid content [23]. Although all four RRTPs effectively inhibited linoleic acid oxidation, their efficacy was not as strong as that of the positive control, VE (IC_50_ of 0.03 mg/mL). Given the synergistic bioactivity typically observed in natural plant polysaccharides, RRTPs could be valuable for producing natural antioxidants.

### 3.4. Anti-Glycation Assay Analysis

Non-enzymatic glycation of proteins, a process implicated in aging, arteriosclerosis, and diabetic complications, progresses through three stages: early, middle, and late [61]. Initially, reducing sugars react with proteins’ amino groups to form transient Schiff bases, which subsequently rearrange into Amadori products. In the middle stage, these products undergo oxidation and dehydration, transforming into α-dicarbonyl compounds such as methylglyoxal, glyoxal, and deoxyglucosones. These compounds further react with amino groups to form advanced glycation end-products (AGEs), which are significant markers and contributors to chronic diseases [62]. In this study, we employed a BSA-fructose model to evaluate the anti-glycosylation efficacy of RRTPs at various glycation stages. During the early stage, Schiff bases and Amadori products form and interact with NBT, producing a detectable colored product at 530 nm [63]. As shown in Figure 5B, RRTPs inhibited Amadori product formation in a dose-dependent manner at concentrations ranging from 0.25 to 2.00 mg/mL. Notably, FD-RRTP demonstrated the strongest inhibition, with an IC_50_ of 2.12 mg/mL, although it was less effective than the reference agent aminoguanidine (AG), which had an IC_50_ of 1.74 mg/mL. Research indicates that the anti-glycosylation properties of polysaccharides, such as those found in arabica coffee husks, are influenced by their degree of esterification and the presence of branched structures (RG-I), characteristics where FD-RRTP particularly excels [45].

During the intermediate phase, α-dicarbonyl compounds form and serve as significant precursors to stable AGEs, facilitating rapid protein cross-linking [63]. As depicted in Figure 5C, the inhibition of these compounds by RRTPs and AG exhibited a dose-dependent trend. Notably, MVD-RRTP showed the most substantial inhibition at 49.06%, although slightly less effective than AG, which exhibited a 51.15% inhibition rate. This difference may be attributed to MVD-RRTP’s lower molecular weight and higher uronic acid content, both of which are pivotal in enhancing its anti-glycosylation efficacy [30,63]. In the final stage, the formation of AGEs was measured. All test samples inhibited AGE formation in a dose-dependent manner, with MVD-RRTP being the most effective among the RRTPs (IC_50_ of 1.80 mg/mL), though less potent than AG (IC_50_ of 0.70 mg/mL). The detection of AGE formation can be effectively conducted using fluorescence spectroscopy. As shown in Figure S2, the fluorescence intensity of the BSA–fructose–RRTPs/AG mixture, after 24 h of incubation at 50 °C, increased significantly when excited at 360 nm, indicating the production of fluorescent AGEs. However, the addition of RRTPs and AG led to a dose-dependent decrease in fluorescence intensity. Additionally, a notable shift in the maximum emission wavelength was observed, suggesting increased polarity around the fluorophore due to the presence of RRTPs. This finding is consistent with previous research, where a BSA–glucose system exhibited a redshift in the maximum emission wavelength following the addition of galangal polysaccharides [64]. The glycosylation studies revealed that all four RRTPs exhibited potent in vitro anti-glycosylation activity. Notably, MVD-RRTP, characterized by its lower molecular weight and higher uronic acid content, demonstrated superior effectiveness. These findings suggest that RRTPs, particularly MVD-RRTP, could serve as viable alternative therapeutic agents for managing glycosylation-related conditions.

### 3.5. In Vitro Hypoglycemic Activity

#### 3.5.1. Inhibitory Activity on α-Glucosidase

**Figure 6:**
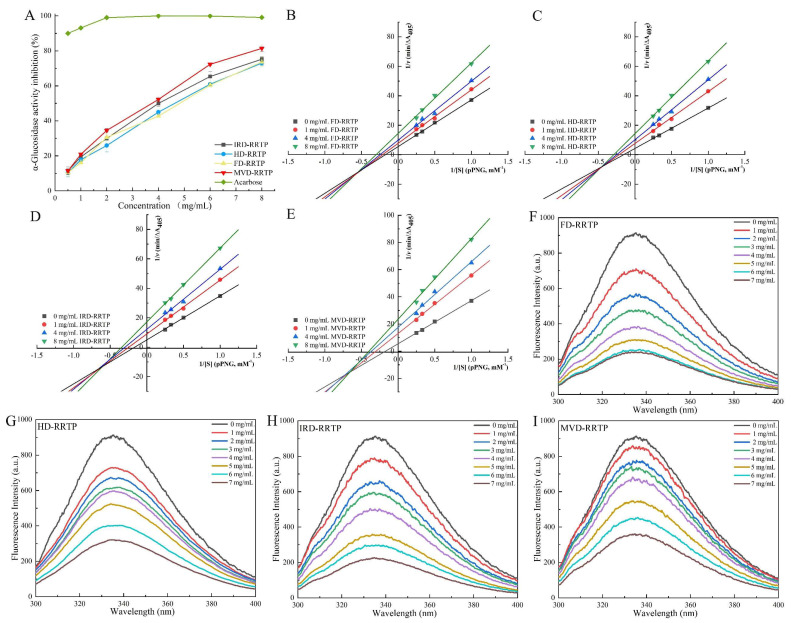
(**A**) α-Glucosidase inhibitory activity of RRTPs; (**B**–**F**) Lineweaver–Burk plots of the reaction of α-glucosidase in the presence of RRTPs; (**G**–**I**) fluorescence emission spectra of α-glucosidase in the presence of various concentrations of RRTPs.

Inhibiting α-glucosidase activity is a recognized strategy for managing diabetes, with numerous studies demonstrating the effectiveness of natural plant polysaccharides in reducing this enzyme’s activity [65,66,67]. As depicted in Figure 6A, RRTPs demonstrated concentration-dependent inhibition of α-glucosidase, ranging from 0.5 to 8.0 mg/mL. Among the tested RRTPs, MVD-RRTP exhibited superior inhibitory effectiveness at all concentrations. The IC_50_ values were 4.14 mg/mL for IRD-RRTP, 4.12 mg/mL for HD-RRTP, 4.11 mg/mL for FD-RRTP, and 3.02 mg/mL for MVD-RRTP, indicating that MVD-RRTP is the most effective inhibitor. Previous research has shown that the -OH and -COOH groups on polysaccharide branches can form strong hydrogen bonds with the active residues of α-glucosidase, leading to significant inhibition [68]. Polysaccharides with higher uronic acid content and lower molecular weight are typically more effective, as these structural characteristics allow greater access to the enzyme’s active sites [65]. This structural advantage likely accounts for MVD-RRTP’s heightened α-glucosidase inhibitory action. To further explore and confirm the potential of RRTPs as α-glucosidase inhibitors, ongoing studies are focusing on the kinetic aspects of their inhibition mechanisms.

#### 3.5.2. Inhibitory Kinetics Analysis

The inhibition type of RRTPs against α-glucosidase was assessed using Lineweaver–Burk plots, as shown in Figure 6B–E. The plots reveal that the Lineweaver–Burk lines for all four RRTPs intersect in the third quadrant. Both *Km* (Michaelis constant) and *Vmax* (maximum velocity) decreased with increasing RRTP concentration, indicative of mixed-type inhibition [66]. This suggests that RRTPs compete with the substrate pNPG for binding sites on α-glucosidase and bind to the enzyme–substrate complex, forming a polysaccharide–α-glucosidase–pNPG ternary complex [69]. Additionally, as noted in Table 3, the inhibition constant for the enzyme alone (*K_i_*) for all RRTPs was higher than the inhibition constant for the enzyme–substrate complex (*K_is_*), indicating a stronger affinity of RRTPs for the α-glucosidase–pNPG complex over free α-glucosidase [67]. This results in decreased *K_m_* and *V_max_*, with *Ki* values larger than *K_is_*, characterizing the inhibition as predominantly non-competitive. Generally, lower *K_i_* and *K_is_* values denote a stronger binding affinity of the inhibitor for the enzyme and the enzyme–substrate complex, respectively, leading to more effective inhibition [70]. As detailed in Table 3, MVD-RRTP demonstrated the lowest *K_i_* (11.74 mg/mL) and *K_is_* (4.16 mg/mL) values among the tested RRTPs, indicating its strong affinity for α-glucosidase. This aligns with the observed effective glucose-lowering inhibition rate, highlighting MVD-RRTP’s potential as an effective natural inhibitor for managing blood glucose levels.

#### 3.5.3. Fluorescence Quenching

Fluorescence quenching is a technique used to monitor reductions in fluorescence quantum yield due to various intermolecular interactions, such as excited state reactions, energy transfer, molecular rearrangements, ground state complex formation, and collisional quenching [20]. The fluorescence emission of proteins is primarily attributed to three aromatic amino acids: tryptophan, phenylalanine, and tyrosine, with tryptophan being the predominant contributor. Thus, the intrinsic fluorescence of α-glucosidase, containing tryptophan residues, can indicate changes in the protein itself and its microenvironment [67]. In this study, fluorescence quenching was employed to investigate the interactions between α-glucosidase and RRTPs. As illustrated in Figure 6F–I, the fluorescence intensity of α-glucosidase decreased with increasing concentrations of RRTPs. Additionally, shifts in fluorescence peak positions were observed following the addition of RRTPs. Specifically, the fluorescence maximum of α-glucosidase shifted from 335.4 nm to 336.4 nm for MVD-RRTP and to 337.6 nm for FD-RRTP, indicating changes in the enzyme’s internal microenvironment. These redshifts have been observed in previous studies and suggest that RRTPs alter the structural or environmental context of α-glucosidase [20,24]. Understanding these interactions is crucial for elucidating how RRTPs affect α-glucosidase activity and stability, offering insights into their potential mechanisms of action.

**Figure 7:**
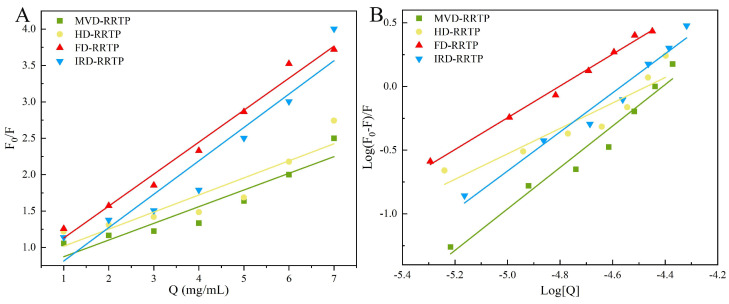
Stern–Volmer plots of α-glucosidase quenched by RRTPs (**A**); plots of log [(F_0_ − F)/F] versus log [Q] for the interaction of RRTPs and α-glucosidase (**B**).

To elucidate the fluorescence quenching mechanism between RRTPs and α-glucosidase, interactions were analyzed at 310.15 K using the Stern–Volmer equation. This equation helps differentiate between static and dynamic quenching mechanisms or a combination of both, commonly observed in enzyme-inhibitor interactions [71]. The quenching constant (*Kq*), derived from the Stern–Volmer plot’s slope, determines the quenching mechanism. A *Kq* value exceeding the maximum scattering collision quenching constant for biopolymers (2.0 × 10^10^ M^−1^s^−1^) typically indicates static quenching [20]. The fluorescence quenching parameters for the four RRTPs are summarized in Table 3, showing *Kq* values of 6.98 × 10^12^, 4.11 × 10^12^, 8.54 × 10^12^, and 3.78 × 10^12^ M^−1^s^−1^ for IRD-RRTP, HD-RRTP, FD-RRTP, and MVD-RRTP, respectively. Since all these values greatly exceed 2.0 × 10^10^ M^−1^s^−1^, it is inferred that the inhibition mechanism of the enzyme by RRTPs is predominantly static quenching. Further insights into the interaction were obtained by analyzing the binding constant (*Ka*) and the number of binding sites (*n*), determined from the static quenching curve (Figure 7). The parameter *n* assesses the number of binding sites available on the enzyme for the inhibitor, while *Ka* evaluates the binding affinity between the inhibitor and the enzyme [72]. The calculated *n* values from Figure 7 for IRD-RRTP (1.53), HD-RRTP (1.00), FD-RRTP (1.25), and MVD-RRTP (1.63) suggest that α-glucosidase possesses one or a similar class of binding sites for these RRTPs [25]. The *Ka* values for IRD-RRTP, HD-RRTP, FF-RRTP, and MVD-RRTP were 1.01 × 10^7^ M^−1^, 2.91 × 10^4^ M^−1^, 9.88 × 10^5^ M^−1^, and 1.50 × 10^7^ M^−1^, respectively. The notably high *Ka* for MVD-RRTP indicates a stronger binding affinity to α-glucosidase, potentially linked to its smaller molecular weight [73]. Comprehensive analysis of these fluorescence parameters reveals that MVD-RRTP exhibits a robust binding capability to the enzyme, corroborating its efficacy in inhibition kinetics and inhibition rate studies.

## 4. Conclusions

This study evaluated the impact of different drying techniques on the physicochemical properties, structural characteristics, and biological activities of polysaccharides extracted from *Rosa roxburghii* Tratt fruit. The findings revealed that drying methods significantly influence the yield, molecular weights, monosaccharide ratios, uronic acid and total sugar contents, gelling properties, particle sizes, and microstructures of the polysaccharides. Biologically, the RRTPs exhibited notable in vitro activities, including anti-linoleic acid oxidation, anti-glycosylation, and α-glucosidase inhibition. Notably, microwave vacuum drying produced polysaccharides (MVD-RRTP) with lower molecular weight and higher uronic acid content, resulting in the most potent anti-linoleic acid oxidation, anti-glycosylation, and inhibitory α-glucosidase effects. These results suggest that *Rosa roxburghii* Tratt fruit polysaccharides, especially those processed using microwave vacuum drying, have potential as valuable dietary supplements and natural agents with antioxidant, antiglycating, and antidiabetic properties. However, to reveal the structure-bioactivity relationship of RRTPs, further purification and structural characterization are required in future studies. This study provides a theoretical foundation for the industrial application of microwave vacuum drying in extracting and processing polysaccharides from *Rosa roxburghii* Tratt fruit, highlighting their potential for enhancing human health.

## Figures and Tables

**Table 1:** Extraction yield, chemical composition, sugar composition, and molecular weight distribution of polysaccharides from RRT dried by different methods.

	IRD-RRTP	HD-RRTP	FD-RRTP	MVD-RRTP
Yield (%)	2.96 ± 0.71 ^d^	3.23 ± 0.55 ^c^	3.75 ± 0.34 ^b^	4.38 ± 0.86 ^a^
Carbohydrate (%)	58.93 ± 2.01 ^c^	57.47 ± 1.42 ^c^	63.90 ± 2.14 ^b^	68.56 ± 1.51 ^a^
Uronic acid (%)	15.78 ± 1.52 ^c^	15.46 ± 1.66 ^c^	18.09 ± 1.16 ^b^	22.16 ± 1.28 ^a^
Protein (%)	1.61 ± 0.22 ^c^	1.57 ± 0.20 ^c^	2.09 ± 0.33 ^b^	2.82 ± 0.15 ^a^
Molecular weight distribution				
*M_w_* (kDa)	164.73 ± 1.22 ^c^	175.01 ± 2.07 ^b^	196.72 ± 1.64 ^a^	145.68 ± 2.54 ^d^
*M_n_* (kDa)	18.04 ± 0.92 ^a^	18.72 ± 1.56 ^a^	20.26 ± 1.46 ^a^	18.44 ± 2.54 ^a^
*M_w_*/*M_n_*	9.15 ± 0.52 ^a^	9.38 ± 0.64 ^a^	9.73 ± 0.60 ^a^	7.95 ± 0.71 ^b^
Monosaccharide composition (molar ratio, %)				
Fuc	0.38 ± 0.29 ^b^	0.42 ± 0.19 ^b^	0.74 ± 0.10 ^a^	0.21 ± 0.11 ^b^
Rha	17.68 ± 0.48 ^a^	17.58 ± 0.48 ^a^	16.98 ± 0.83 ^ab^	15.83 ± 1.23 ^b^
Ara	6.45 ± 0.27 ^b^	6.51 ± 0.35 ^b^	6.35 ± 0.31 ^b^	8.99 ± 1.35 ^a^
Gal	35.69 ± 1.10 ^b^	35.44 ± 0.14 ^b^	39.23 ± 1.81 ^a^	34.58 ± 0.34 ^b^
Glc	4.10 ± 1.17 ^a^	4.87 ± 0.40 ^a^	4.26 ± 0.42 ^a^	3.54 ± 0.69 ^b^
Xyl	1.56 ± 1.01 ^a^	0.81 ± 0.32 ^b^	1.58 ± 0.43 ^a^	0.70 ± 0.11 ^b^
Man	3.15 ± 1.43 ^ab^	4.91 ± 1.21 ^a^	1.78 ± 1.52 ^b^	1.17 ± 0.15 ^b^
GalA	29.52 ± 2.70 ^b^	28.82 ± 0.99 ^bc^	27.47 ± 1.45 ^c^	33.79 ± 1.64 ^a^
GlcA	1.48 ± 0.06 ^a^	1.64 ± 0.24 ^a^	1.61 ± 0.31 ^a^	1.20 ± 0.19 ^a^
HG (%)	11.84 ± 1.07 ^b^	10.49 ± 1.67 ^b^	11.24 ± 1.16 ^b^	17.96 ± 1.87 ^a^
RG-I (%)	77.49 ± 2.10 ^ab^	79.53 ± 2.34 ^a^	76.12 ± 1.23 ^b^	75.23 ± 3.88 ^b^
(Ara + Gal)/Rha	2.38 ± 0.06 ^b^	2.69 ± 0.07 ^a^	2.33 ± 0.05 ^b^	2.75 ± 0.06 ^a^
Rha/GalA	0.60 ± 0.07 ^a^	0.62 ± 0.04 ^a^	0.61 ± 0.03 ^a^	0.48 ± 0.11 ^b^
Linearity	0.47 ± 0.05 ^a^	0.41 ± 0.03 ^b^	0.47 ± 0.02 ^a^	0.56 ± 0.12 ^a^
DE (%)	57.02 ± 0.97 ^b^	58.23 ± 3.42 ^a^	59.12 ± 2.35 ^a^	59.31 ± 0.10 ^a^

Values are expressed as mean ± SD (*n* = 3). Values in the same row with different letters indicate significant differences (*p* < 0.05). HG/% = GalA − Rha. RG-I/% = 2 Rha + Gal + Ara. Linearity = GalA/(Fuc + Rha + GlcA + Ara + Gal + Xyl).

**Table 2:** DSC curve parameters of polysaccharides from RRT dried by different methods.

	IRD-RRTP	HD-RRTP	FD-RRTP	MVD-RRTP
*Tm* (°C)	121.33 ± 1.16 ^b^	123.58 ± 5.88 ^b^	148.72 ± 3.06 ^a^	136.49 ± 1.41 ^ab^
Δ*Hm* (J/g)	141.85 ± 43.51 ^b^	112.46 ± 28.33 ^c^	183.89 ± 40.94 ^a^	137.58 ± 29.55 ^b^
*To* (°C)	237.49 ± 2.69 ^c^	241.57 ± 3.90 ^bc^	247.58 ± 5.04 ^ab^	251.65 ± 1.69 ^a^
*Tg* (°C)	257.80 ± 0.72 ^a^	258.91 ± 3.28 ^a^	256.25 ± 0.81 ^a^	258.37 ± 2.68 ^a^
Δ*Hg* (J/g)	30.18 ± 14.61 ^a^	5.03 ± 2.39 ^b^	21.67 ± 6.72 ^a^	3.21 ± 1.80 ^b^

Values are expressed as mean ± SD (*n* = 3). Values in the same row with different letters indicate significant differences (*p* < 0.05).

**Table 3:** Inhibitory kinetic parameters of α-glucosidase after interaction with different RRTPs.

Sample	Concentration (mg/mL)	*K_m_* (mM)	*V_max_* (∆A405/min)	*K_i_* (mg/mL)	*K_is_* (mg/mL)	*K_q_* (M^−1^ s^−1^)	KSV (M−1)	*K_α_* (M^−1^)	n
IRD-RRTP	0	6.02	0.20	13.81	4.49	6.98 × 10^12^	6.98 × 10^4^	1.01 × 10^7^	1.53
	1	4.07	0.11						
	4	3.35	0.08						
	8	2.93	0.06						
HD-RRTP	0	6.48	0.24	12.09	4.64	4.11 × 10^12^	4.11 × 10^4^	2.91 × 10^4^	1.00
	1	4.65	0.13						
	4	4.02	0.10						
	8	3.47	0.07						
FD-RRTP	0	6.95	0.22	18.08	4.92	8.54 × 10^12^	8.54 × 10^4^	9.88 × 10^5^	1.25
	1	4.73	0.13						
	4	4.06	0.10						
	8	3.33	0.07						
MVD-RRTP	0	5.80	0.18	11.74	4.16	3.78 × 10^12^	3.78 × 10^4^	1.50 × 10^7^	1.63
	1	3.30	0.08						
	4	2.75	0.06						
	8	2.25	0.04						

## Data Availability

The original contributions presented in the study are included in the article, further inquiries can be directed to the corresponding author.

## Supplementary Materials

The following supporting information can be downloaded at: https://www.mdpi.com/article/10.3390/foods13152417/s1, Figure S1: DSC thermograms of RRTPs; Figure S2: The fluorescence spectra of AGEs in the presence of increasing concentrations of AG, IRD-RRTP, HD-RRTP, FD-RRTP and MVD-RRTP.
