# Supplementary material for: Effects of Different Drying Methods on the Structural Characteristics and Multiple Bioactivities of Rosa roxburghii Tratt Fruit Polysaccharides
Source: Foods. 2024 Jul 30;13(15):2417. doi: 10.3390/foods13152417 (PMC11312052; doi:10.3390/foods13152417)
Supplement: Supplementary file 1 [file foods-13-02417-s001.zip › foods-3109777-supplementary.pdf]

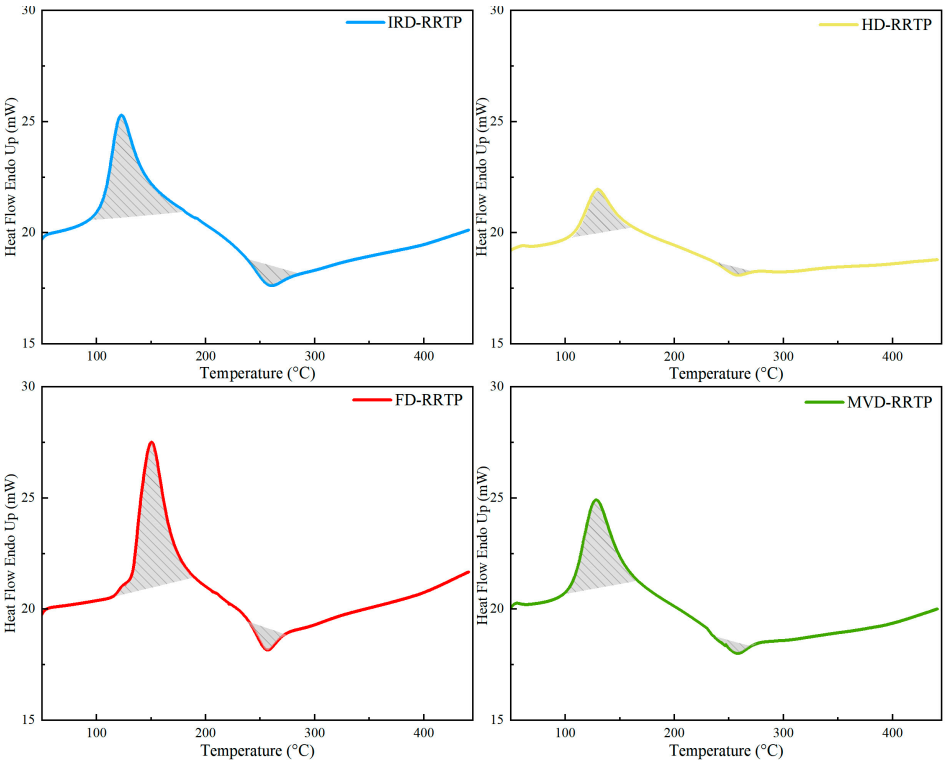

Figure S1. DSC thermograms of RRTPs.

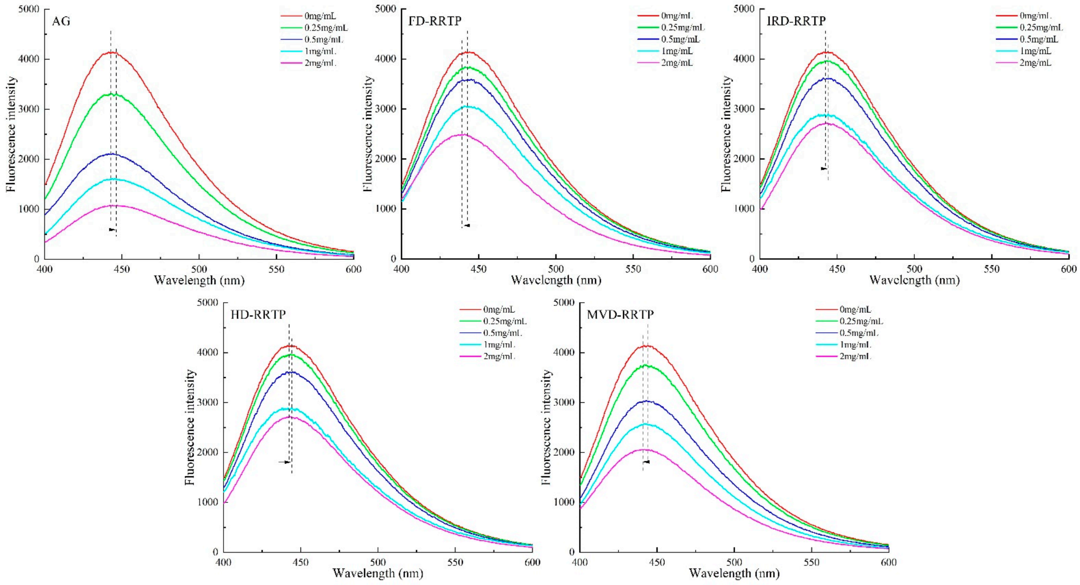

Figure S2. The fluorescence spectra of AGEs in the presence of increasing concentrations of AG, IRD-RRTP, HD-RRTP, FD-RRTP and MVD-RRTP.
